# Clinical and molecular report of c.1331 + 1G > A mutation of the *AAAS* gene in a Moroccan family with Allgrove syndrome: a case report

**DOI:** 10.1186/s12887-018-1161-4

**Published:** 2018-06-04

**Authors:** H. Berrani, T. Meskini, M. Zerkaoui, H. Merhni, S. Ettair, A. Sefiani, N. Mouane

**Affiliations:** 1 Pediatrics III, Children’s Hospital of Rabat, University Mohammed V, Belarbi El Alaoui Avenue, 6203 Rabat, PB Morocco; 2 Nutrition and Food Science Departments, Faculty of Medicine and Pharmacy, Mohammed V University-Rabat, Belarbi El Alaoui Avenue, 6203 Rabat, Morocco; 3 Medical Genetics Institute, Institut National d’Hygiène, University Mohammed V, Rabat, Morocco

**Keywords:** Allgrove syndrome, C.1331 + 1G > A mutation, *AAAS* gene

## Abstract

**Background:**

Allgrove syndrome is a rare autosomal recessive disorder characterized by the triad of achalasia, alacrimia and adrenal insufficiency. It is caused by the mutations of the *AAAS* gene located on chromosome 12q13. The c.1331 + 1G > A mutation is one of the most common described in North Africa including Tunisia, Algeria and Libya. We report here the clinical and genetic profile of a Moroccan family with Allgrove syndrome.

**Case presentation:**

A Moroccan sister and brother born to consanguineous parents were found, at the ages of twelve and fifteen months old respectively, to have alacrimia and isolated glucocorticoid deficiency. Later, they developed achalasia whereupon Allgrove syndrome was diagnosed clinically and confirmed by DNA sequencing which revealed a c.1331 + 1G > A mutation in the *AAAS* gene.

**Conclusion:**

This finding reinforces previous studies in demonstrating the geographic expansion of the ancestral mutation c.1331 + 1G > A in North African patients and thus enabling targeted genetic counseling. To the best of our knowledge, this is the first report of the *AAAS* gene mutation in Moroccan patients.

## Background

The triple association of achalasia, alacrimia and adrenal insufficiency characterizes the Allgrove syndrome (AS, OMIM 231550), also known as the triple A syndrome [1–3]. The more recent recognition of a fourth component - autonomic dysfunction, in association with motor neuropathy, sensory disorder, mental retardation and similar neurologic diseases, has given rise to the term “4 A syndrome” [4, 5]. AS is a progressive disorder with various clinical manifestations The syndrome usually occurs in the first decade of life but cases of late onset in adulthood have been reported [6]. The *AAAS* gene is located on chromosome 12q13 and encodes the ALADIN protein (alacrima, achalasia, adrenal insufficiency and neurological disorder) [7]. The pathogenic gene has a ubiquitous expression in the human tissues with a particularly high expression in the adrenal gland, gastrointestinal tract and brain [8]. AS has been reported from different parts of the world. The c.1331 + 1G > A mutation is one of the most common mutations described in the literature particularly in North Africa, having been identified in Tunisian, Algerian and Libyan populations recently [9]. According to an extensive literature review and to the best of our knowledge, we describe here the first case of an *AAAS* gene mutation, namely the c.1331 + 1G > A genotype, to be reported in Morocco.

## Case presentation

Case 1: A five-year-old Moroccan girl was diagnosed clinically as having AS at 12 months. Her apparently healthy parents were third cousins. She was born by normal vaginal delivery after a full-term pregnancy with a normal birth weight of 3800 g. From the age of 5 months she had a history of asthenia, anorexia and vomiting. From 12 months, she had generalized hyperpigmentation of the skin and failure to thrive. She had not been able to produce tears since infancy. Physical examination at this time showed height 67 cm (− 2.84 SD), weight 8.300 g (− 1.27 SD), blood pressure 84/43 mmHg. No abnormality was found in the heart, lungs, abdomen, nervous system and external genitalia. Baseline investigations revealed normal full blood count, serum creatinine and electrolytes. Basal serum cortisol at 8 AM was unrecordable at < 0.3 nmol/L while plasma adrenocorticotropic hormone (ACTH) was markedly elevated at 228.8 pmol/L. The results of a bilateral Schirmer test confirmed the diagnosis of alacrima. Hydrocortisone therapy replacement was started at 10 mg/m^2^/day with artificial tears and topical vitamin A. At this time, esophagography showed no abnormality. At follow up, aged 2 years, the patient presented with high blood pressure - 120/70 mmHg. Plasma aldosterone was 170 pmol/l (reference range 22–477) and plasma renin activity was 359.7mUI/l with a normal aldosterone to renin ratio. Echocardiography was normal. Autonomic dysfunction and hence the 4 A syndrome was therefore diagnosed. After 10 months of the treatment with captopril, the blood pressure normalized at 90/50 mmHg and antihypertensive treatment was stopped. At this time, the patient presented with perioral cyanosis and cold extremities and was found to have hyponatraemia (plasma sodium 126 mmol/L) with high urine sodium (35 mmol/24 h). Mineralocorticoid deficiency was diagnosed and treated with fludrocortisone 50 μg per day. Partial loss of primary teeth was noted from 2 years of age at which time esophagography showed a dilated esophagus with distal narrowing. Manometry was not performed in view of the patient’s age. Following the diagnosis of achalasia, the patient underwent surgery with esophago-cardiomyotomy and fundoplication. She became symptom free for about 19 months, but the dysphagia recurred and was treated with balloon dilatation of the esophagus. A year later the symptoms recurred and balloon dilatation was again performed but without relief of symptoms on this occasion. High resolution manometry showed normal lower esophageal sphincter (LES) resting pressure (29 mmHg) with incomplete relaxation, prolonged integrated relaxation pressure (IRP) (27 mmHg) and aperistalsis. By the age of 3 years and six months, her weight had become static at 10 kg (− 2.5 SD) and a gastrostomy tube was inserted. Currently the patient is aged five years, weight 15 kg (− 1.5 SD) and height 97 cm (− 2 SD).

**Fig. 1:**
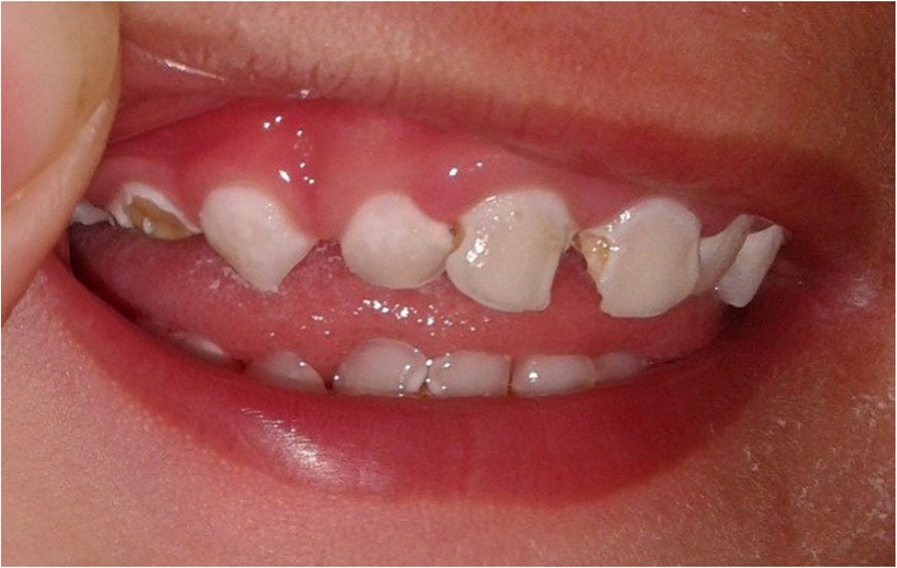
Patient 2 with Allgrove Syndrome showing partial loss of primary dentition

**Fig. 2:**
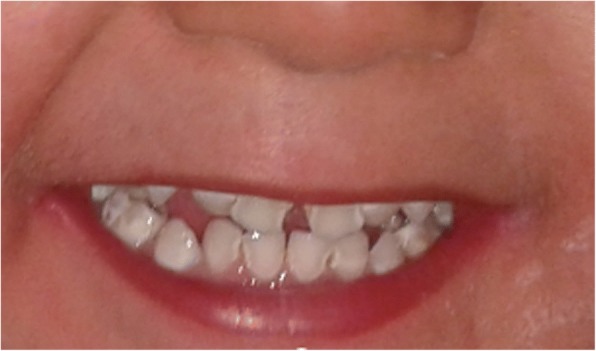
Patient 2 with Allgrove Syndrome showing long philtrum and narrow upper lip

**Fig. 3:**
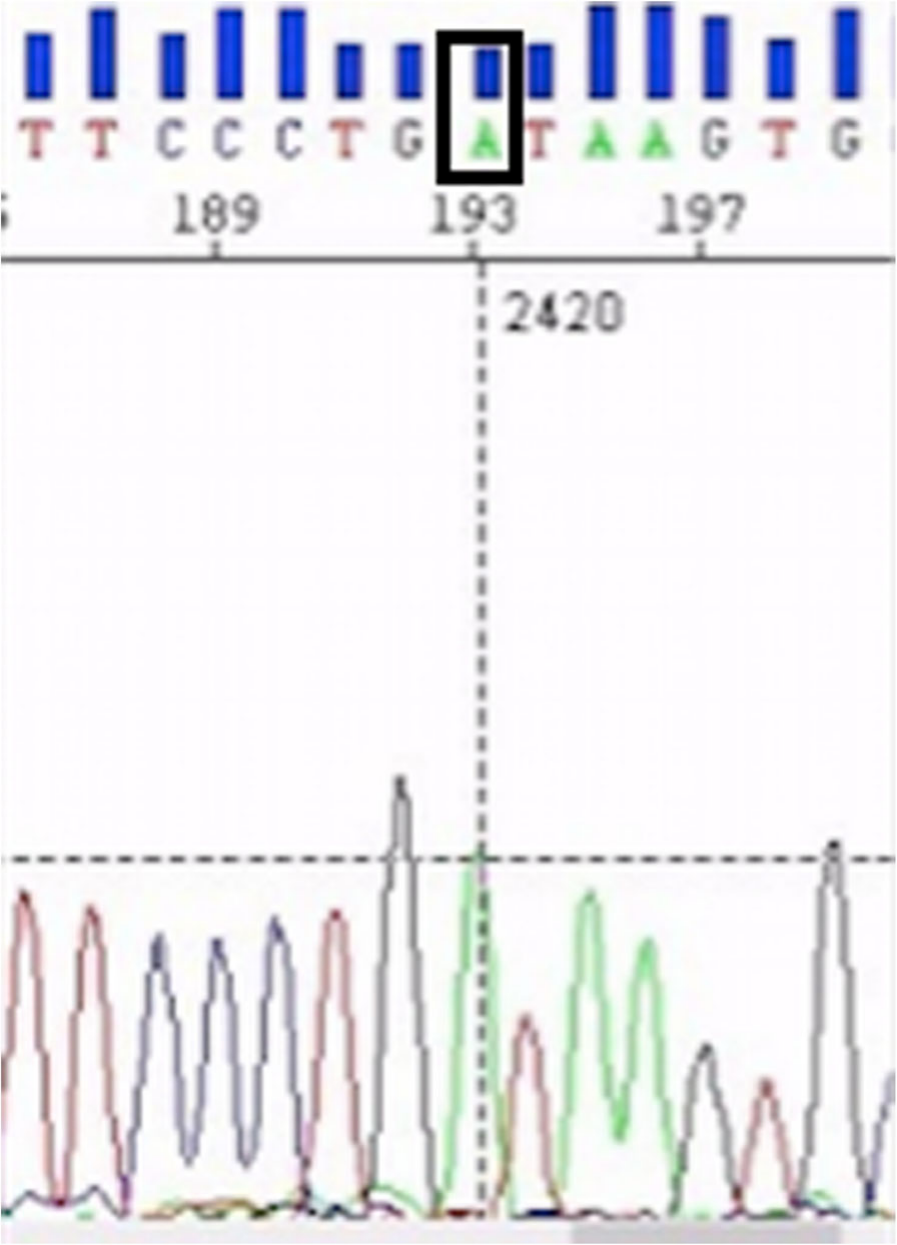
DNA sequencing showing a c.1331 + 1G > A mutation of the *AAAS* gene

Case 2: The brother of Case 1 is aged 2.6 years and was born by normal vaginal delivery at term, birth weight normal at 3400 g. His parents noted no tears while crying from birth. From 9 months there was a history of generalized weakness, asthenia and anorexia, with progressive hyperpigmentation of the skin for 3 months. On admission at the age of 15 months, height was 75 cm (− 2 SD), weight 9 kg (− 2 SD) and blood pressure was 83/50 mmHg. A long philtrum and narrow upper lip were observed (Fig. 1). Hyperpigmentation was noted over most of the body. Examination of the nervous system and external genitalia was normal. Baseline investigations revealed normal complete blood count, serum creatinine and electrolytes with serum sodium 139 mmol/L potassium 4.3 mmol/L and urine sodium 20 mmol/24 h. Basal serum cortisol at 8 am was unrecordable at < 0.3 nmol/L with markedly elevated ACTH at 416 pmol/L. The results of a bilateral Schirmer test were 4 mm, confirming the diagnosis of severe hypolacrima. Esophagography was normal at this time. AS was diagnosed and treatment with hydrocortisone, artificial tears and topical vitamin A was started. At 2 and a half years the boy developed dysphagia and vomiting. Esophagography now showed achalasia of the lower esophagus at the cardia. He also had partial loss of primary dentition (Fig. 2). Genetic testing confirmed a c.1331 + 1G > A homozygote mutation of the *AAAS* gene (Fig. 3).

## Discussion and conclusion

Allgrove syndrome can be considered as a multisystem disorder, which can be life-threatening when diagnosis is delayed. The features of AS are ACTH resistant adrenal insufficiency, achalasia and alacrimia. Review of literature reveals alacrima as the earliest and most consistent clinical sign of AS and was indeed the initial symptom reported in our patients. Thus, when a child presents with alacrima Allgrove syndrome should be suspected, even if the other typical clinical features are absent, and particularly when the patient is of North African origin. Adrenal insufficiency is also an early manifestation of AS, and may present with severe hypoglycemia or hypotension, which may lead to sudden death during childhood [10]. Less frequently, adrenal insufficiency may present with chronic vomiting, hyperpigmentation, or developmental delay [11]. Mineralocorticoid production is generally preserved in AS but can be impaired in 15% of patients [11]. Both of our patients showed isolated glucocorticoid deficiency initially but then developed mineralocorticoid deficiency. The esophageal dysfunction in AS features absence of peristalsis within the body of the esophagus with a relaxation defect in the gastro-esophageal sphincter and secondary dilatation in the proximal esophagus. A severe form of achalasia was noted in our first patient, managed by repeated balloon dilatation after surgery had proved to be of only temporary benefit only, followed by insertion of gastrostomy tube when dilatation was no longer effective. Neurological symptoms may manifest in certain subgroups of patients with AS who show a less severe and more chronic course of the disease [12]. Currently our first patient has presented with autonomic dysfunction and was thus diagnosed as having 4 A syndrome, however the second patient has not exhibited yet any neurological symptoms. Our patients also presented with partial loss of their primary dentition which occurried at the same age as gastro-esophageal disease was diagnosed. We have excluded other possible causes of this dental loss, such as trauma, local infection or dental caries. Two cases of two siblings with AS and premature loss of permanent teeth were reported by Palka et al. [13]. These authors speculated that tooth loss could be an additional feature of the multisystemic disorder. Our patients also had some dysmorphic features such as long philtrum and thin upper lip which may be relevant to the genotype.

AS is caused by a mutation in the *AAAS* gene, located on chromosome 12q13. More than 70 mutations have been described in patients from different parts of the world. The c.1331 + 1G > A mutation is one of the most frequent mutations of the *AAAS* gene in the world and the most frequent mutation in North Africa [9] being found Algerian and Tunisian families [14] and recently in Libyan families [9]. This frequent mutation seems to be inherited from a common ancestor and spread in North African populations [9] and it is of note that the c.1331 + 1G > A mutation was found in our family. To our knowledge, this is the report of a c.1331+1G>A mutation in a Moroccan family and it is this mutation which should be specifically looked for in future patients from Maghreb countries with a clinical diagnosis of AS.

## Abbreviations

ACTH: Plasma adrenocorticotropic hormone
AS: Allgrove syndrome
IRP: Integrated relaxation prolonged
LES: Lower esophageal sphincter
